# Transient Receptor Potential Channel 1 Potentially Serves as a Biomarker Indicating T/TNM Stages and Predicting Long-Term Prognosis in Patients With Renal Cell Carcinoma

**DOI:** 10.3389/fsurg.2022.853310

**Published:** 2022-04-25

**Authors:** Liang Chen, Guang Shan, Minghuan Ge, Huijun Qian, Yue Xia

**Affiliations:** Department of Urology, Renmin Hospital of Wuhan University, Wuhan, China

**Keywords:** transient receptor potential channel 1, renal cell carcinoma, T stage, TNM stage, overall survival

## Abstract

**Background:**

Transient receptor potential channel 1 (TRPC1) regulates the progression of several cancers, but its clinical implication in renal cell carcinoma (RCC) has not been explored yet. This study aimed to investigate the correlation of TRPC1 with clinical characteristics and prognosis in patients with RCC.

**Methods:**

Totally, 177 patients with primary RCC who received surgical resection were retrospectively screened. Their tumor and paired adjacent tissue specimens were retrieved to assess TRPC1 mRNA expression using RT-qPCR and TRPC1 protein expression using immunohistochemistry (IHC).

**Results:**

Both TRPC1 IHC score and TRPC1 mRNA expression were elevated in RCC tissue than in adjacent tissue (both *P* < 0.001). Meanwhile, both TRPC1 IHC score and TRPC1 mRNA expression in tumor were associated with higher T stage (both *P* = 0.02) and TNM stage (*P* = 0.009, *P* = 0.003, respectively). However, no correlation was found in tumor TRPC1 IHC score or TRPC1 mRNA expression with other tumor properties (all *P* > 0.05). Besides, the 3-, 5-, and 7-year overall survival (OS) were 81.4, 68.6, and 60.2%, respectively in patients with high tumor TRPC1 protein, while they were 89.3, 82.7, and 76.7%, respectively in patients with low tumor TRPC1 protein. High (vs. low) TRPC1 protein in the tumor was associated with shorter OS (*P* = 0.017), while high (vs. low) TRPC1 mRNA in the tumor was not correlated with OS (*P* = 0.144). By the forward stepwise method, TRPC1 protein expression independently predicted poor OS (*P* = 0.01, hazard ratio = 2.052).

**Conclusion:**

TRPC1 serves as a potential biomarker reflecting tumor features and long-term survival profile in patients with RCC.

## Introduction

Renal cell carcinoma (RCC) is a kidney cancer with yearly mortality exceeding 140,000 cases reported by the WHO in the year 2019, which is consisted of various histological subtypes, such as clear cell, papillary, and chromophobe subtypes (1–4). Many patients with RCC present with complications of hypercalcemia, fever, or paraneoplastic syndrome (5, 6). Currently, advancement in surveillance has been achieved to help early diagnosis of RCC, and treatment methods, such as nephrectomy, nephron-sparing tumor excision, ablation, and systemic drug therapy, have greatly improved the 5-year overall survival (OS) of patients with RCC. However, the prognosis of RCC is still poor among patients who develop metastatic disease. Even for patients without metastasis or diagnosed at early-stage, their outcomes after treatment differ sharply due to tumor heterogeneity (7–10). Besides, donor-transmitted cancer after kidney transplantation has been increasingly reported in recent years, among which RCC ranks as one of the most common types (11); while there lack potential biomarkers to identify and predict the risk of RCC in both donors and recipients under this scenario. Therefore, finding out new biomarkers may help the surveillance of RCC and improve the management of patients with RCC.

Transient receptor potential channel 1 (TRPC1) is a key member of the transient receptor potential (TRP) protein superfamily and serves as a potential regulator of store-operated Ca^2+^ entry (SOCE) pathways (12). It is reported that TRPC1 plays a vital role in various cancers (12). For instance, TRPC1 modulates the phosphatidylinositol 3-kinase/protein kinase B (PI3K/AKT) pathway and epithelium-mesenchymal transformation (EMT), subsequently influencing cell proliferation and migration of breast cancer cells (13). Clinically, TRPC1 is regarded as a biomarker for the prognosis of cancer patients, such as breast cancer and esophageal squamous cell carcinoma (ESCC) (13–15). TRPC1 also plays an important role in regulating renal fibrosis, a critical signal of RCC initiation (16). Based on the above-mentioned information, we hypothesized that TRPC1 might be a potential biomarker for RCC.

In this study, we detected the TRPC1 protein expression by immunohistochemistry (IHC) and the TRPC1 mRNA expression by RT-qPCR in the RCC tissue and adjacent tissue, and subsequently investigated their linkages with tumor features and prognosis in patients with RCC.

## Methods

### Patients

This study was approved by the Institutional Review Board, and the study retrospectively screened 177 patients with primary RCC who received surgical resection in our hospital between January 2011 and December 2015. The main screening criteria were set as follows: (i) histologically confirmed as RCC; (ii) clear cell subtype RCC (to avoid potential interruption); (iii) 18–80 years old; (iv) received surgical resection; (v) had available tumor tissue specimens and paired adjacent tissue (normal renal cortex) specimens for IHC analysis; (vi) had complete preoperative clinical features and at least one follow-up data able to perform survival analysis. Patients with history or complicated with other tumors were excluded from the study.

### Data and Specimen Collection

Clinical features of the patients with RCC were abstracted from the medical documents, which included age, gender, Eastern Cooperative Oncology Group Performance Status (ECOG PS) score, tumor location, tumor size, pathological grade, and tumor-node-metastasis (TNM) stage (2011 version). Besides, the survival data of all patients were obtained. The final date of the follow-up recording was 31 January 2021. OS was calculated based on the survival data. For specimen collection, tumor and paired adjacent tissue specimens from all patients were fixed in formalin and embedded in paraffin (FFEP) for IHC assay. In addition, tumor and paired adjacent tissue specimens from 93 of all patients were frozen in liquid nitrogen to carry out the RT-qPCR assay.

### IHC Assay

Immunohistochemistry assay was used to assess TRPC1 protein expression, and the procedure was described in a previous study (13). In brief, the FFEP samples were cut into 4-μm slices. Then, the slides were deparaffinized with xylene, rehydrated with gradient ethanol, heated in 0.01 mol/L sodium citrate buffer for antigen retrieval, and quenched with fresh 3% hydrogen peroxide to inhibit the activity of endogenous peroxidase. Sequentially, the slices were incubated using goat anti-TRPC1 antibody (dilution 1:150; Abcam, Waltham, MA, USA) as primary antibody, then incubated using rabbit anti-goat immunoglobulin G (IgG) (H&L) (dilution 1:2000; Abcam, Waltham, MA, USA) as the secondary antibody. Finally, 3,3'-diaminobenzidine (DAB, Sangon Biotech Co., Ltd., Shanghai, China) and hematoxylin were used for slice staining.

After staining, IHC results were evaluated by two pathologists who were blind to the patients' clinical or pathological data using a light microscope by a semi-quantitatively method according to intensity and density of stained cells. The IHC score was the average of the two pathologist scores, and the consistency coefficient of the two pathologists scoring was 0.917. The intensity was scored as four grades: 0 (negative), 1 (weak), 2 (moderate), and 3 (strong). The density was scored as five grades: 0 (0%), 1 (1–25%), 2 (26–50%), 3 (51–75%), and 4 (76–100%). The final score of the IHC assay was a product of the intensity score and the density score. According to the IHC score, TRPC1 protein expression was classified as high expression (final IHC score > 3) and low expression (final IHC score ≤ 3) based on a previous study (17).

### RT-qPCR Assay

The RT-qPCR assay was used to assess TRPC1 mRNA expression. The quantitative analysis of TRPC1 expression was carried out by the 2^−Δ*ΔCt*^ method with GAPDH as the reference gene. The primers were designed referring to a previous study (18). The sample was treated by TRIzol™ Reagent (Thermo Fisher Scientific, Waltham, MA, USA) to extract total RNA, then submitted to perform reverse transcription using iScript™ cDNA Synthesis Kit (Bio-Rad, Hercules, CA, USA). Thereafter, qPCR was conducted using KOD SYBR^®^ qPCR Mix (Toyobo, Osaka, Kansai, Japan). After RT-qPCR assay, the relative expression of TRPC1 mRNA in tumor tissue was classified based on its median value (2.642) into high expression (≥ 2.642) and low expression (< 2.642).

### Statistical Analysis

To analyze patients' data, SPSS 24 (IBM Corp., Armonk, NY, USA) was applied, and GraphPad Prism 7.01 (GraphPad Software Inc., San Diego, CA, USA) was used to construct graphs. Comparisons of TRPC1 expression between tumor tissue specimens and adjacent tissue specimens were evaluated using the paired-samples *t*-test and the Wilcoxon signed-rank test. Correlations between TRPC1 expression and clinical features were assessed using the Chi-square test, Fisher's exact test, and the linear by linear test. OS was elucidated using the Kaplan–Meier curves and assessed using the log-rank test. Cox's proportional hazard regression model analysis was applied for prognostic analysis. Statistical significance was concluded if a two-sided *P*-value was less than 0.05.

## Results

### Clinical Characteristics

A total of 177 patients with primary RCC who received surgical resection were recruited in the present study, with their clinical characteristics summarized in Table 1. In brief, the mean age of patients with RCC was 58.8 ± 11.4 years. The numbers of patients with RCC who were women and men were 55 (31.1%) and 122 (68.9%), respectively. The tumor location of 86 (48.6%) patients was on the right, whereas that of 91 (51.4%) patients was on the left. Regarding the pathological grade, 81 (45.8%), 73 (41.2%), and 23 (13.0%) patients were at G1, G2, and G3, respectively. Besides, the median (IQR) tumor size was 5.5 (4–8) cm. Concerning the T stage, there were 63 (35.6%), 61 (34.5%), 35 (19.8%), 7 (4%), and 11 (6.2%) patients at stage T1a, T1b, T2a, T2b, and T3, respectively. Moreover, 162 (91.5%) patients presented with the N0 stage, while 15 (8.5%) patients presented with the N1 stage. Furthermore, 116 (65.5%), 39 (22.0%) and 22 (12.4%) patients were at TNM stages I, II, and III, respectively.

**Table 1:** Clinical features.

Items	RCC (*N* = 177)
Age (years), mean ± SD	58.8 ± 11.4
**Gender, No. (%)**	
Female	55 (31.1)
Male	122 (68.9)
**ECOG PS score, No. (%)**	
0	143 (80.8)
1	34 (19.2)
**Tumor location, No. (%)**	
Right	86 (48.6)
Left	91 (51.4)
**Pathological grade, No. (%)**	
G1	81 (45.8)
G2	73 (41.2)
G3	23 (13.0)
Tumor size (cm), median (IQR)	5.5 (4.0–8.0)
**T stage, No. (%)**	
T1a	63 (35.6)
T1b	61 (34.5)
T2a	35 (19.8)
T2b	7 (4.0)
T3	11 (6.2)
**N stage, No. (%)**	
N0	162 (91.5)
N1	15 (8.5)
**TNM stage, No. (%)**	
I	116 (65.5)
II	39 (22.0)
III	22 (12.4)

*RCC, renal cell carcinoma; SD, standard deviation; ECOG PS, Eastern Cooperative Oncology Group Performance Status; IQR, interquartile range; TNM, tumor-node-metastasis*.

### TRPC1 Expression in RCC Adjacent and Tumor Tissue

**Figure 1:**
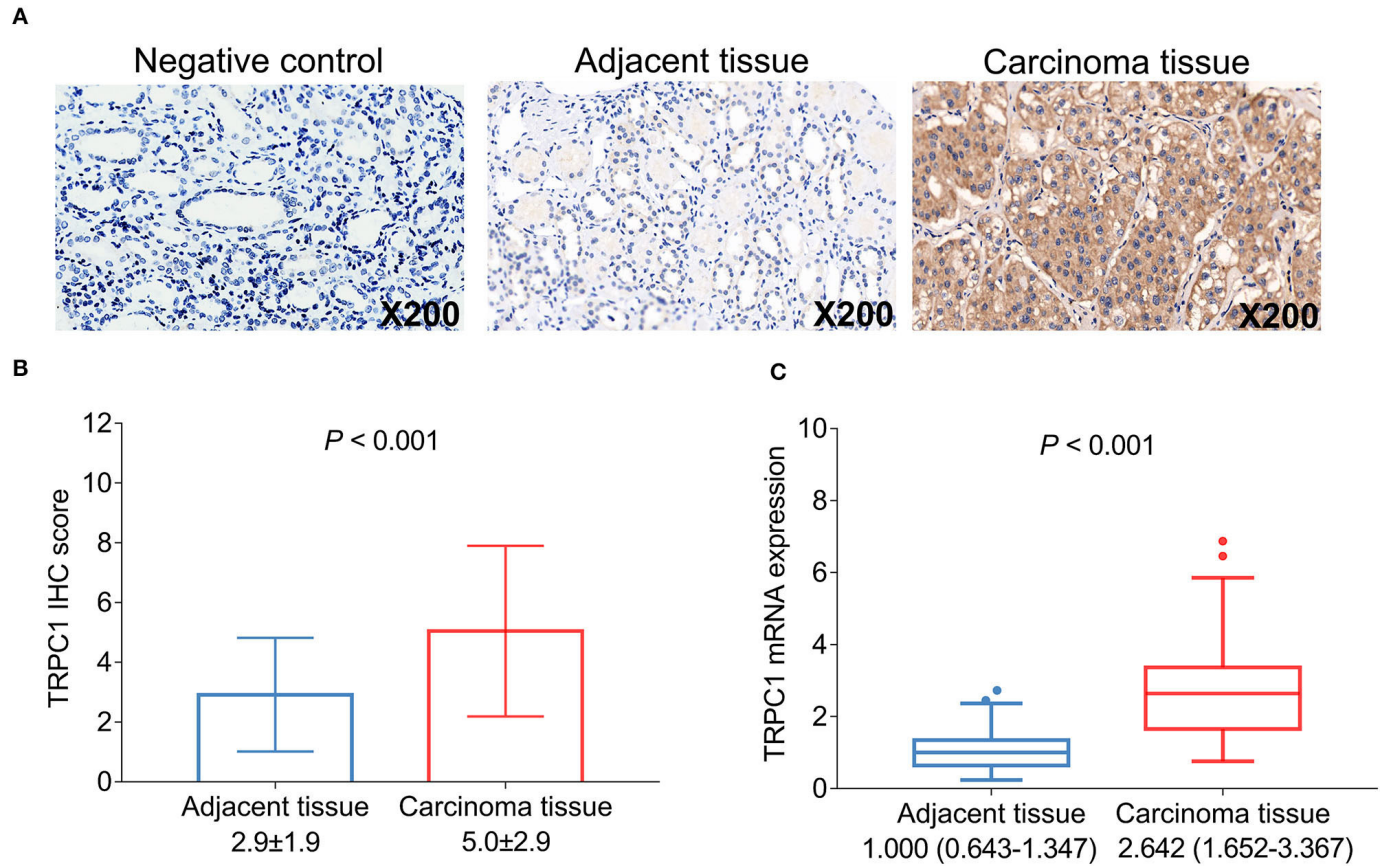
Transient receptor potential channel (TRPC1) expression in patients with renal cell carcinoma (RCC). Samples of TRPC1 immunohistochemistry (IHC) staining in the negative control, adjacent and tumor tissues **(A)**; comparison of TRPC1 IHC score **(B)** and TRPC1 mRNA expression **(C)** between adjacent and tumor tissues. TRPC1, transient receptor potential channel 1; IHC, immunohistochemistry; mRNA, messenger RNA; RCC, renal cell carcinoma.

Transient receptor potential channel 1 was validated in control tissue and detected by IHC assay in the tumor tissue and the adjacent tissue (Figure 1A). After analysis, the TRPC1 IHC score was increased in the tumor tissue compared with the adjacent tissue (5 ± 2.9 vs. 2.9 ± 1.9, *P* < 0.001) (Figure 1B); TRPC1 mRNA expression was higher in the tumor tissue than in the adjacent tissue (2.642 (1.652–3.367) vs. 1 (0.643–1.347), *P* < 0.001) (Figure 1C). In addition, TRPC1 mRNA expression and TRPC1 IHC score were closely correlated with each other in tumor and adjacent tissues, respectively (both *P* < 0.001) (Supplementary Figures 1A,B).

### Correlation of TRPC1 Expression With Tumor Features

**Figure 2:**
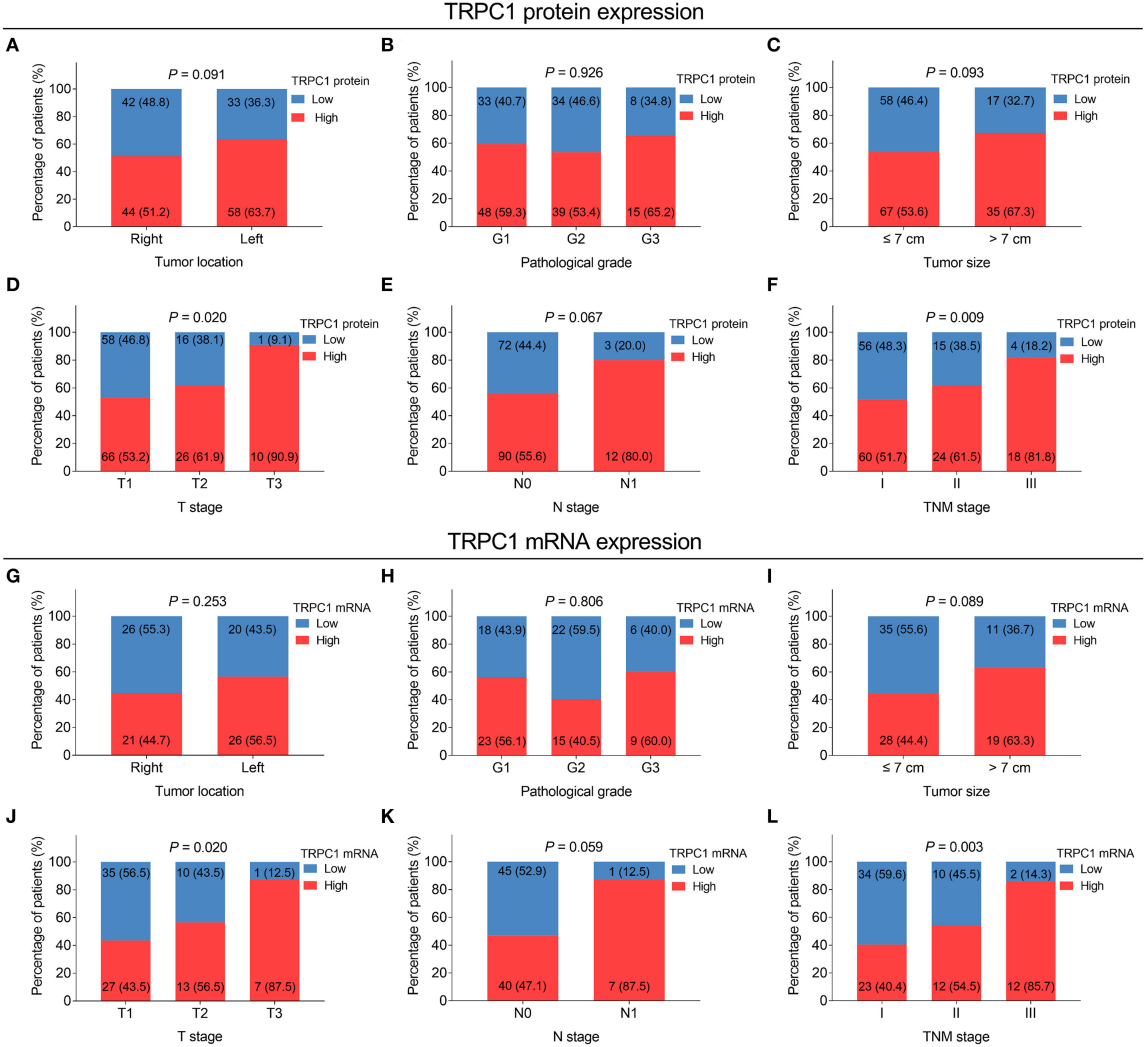
Correlation of TRPC1 expression with tumor features in patients with RCC. Correlation of TRPC1 protein expression with tumor location **(A)**, pathological grade **(B)**, tumor size **(C)**, T stage **(D)**, N stage **(E)**, and tumor-node-metastasis (TNM) stage **(F)**; association of TRPC1 mRNA expression with tumor location **(G)**, pathological grade **(H)**, tumor size **(I)**, T stage **(J)**, N stage **(K)**, and TNM stage **(L)**. TRPC1, transient receptor potential channel 1; TNM, tumor-node-metastasis; mRNA, messenger RNA; RCC, renal cell carcinoma.

Transient receptor potential channel 1 protein expression was correlated with more advanced T stage (*P* = 0.02) and TNM stage (*P* = 0.009). However, no correlation was found in TRPC1 protein expression with tumor location, pathological grade, tumor size, or N stage (all *P* > 0.05) (Figures 2A–F). TRPC1 mRNA expression was associated with elevated T stage (*P* = 0.02) and TNM stage (*P* = 0.003) as well. However, no association was discovered in TRPC1 mRNA expression with other tumor properties (all *P* > 0.05) (Figures 2G–L).

### Association of Tumor TRPC1 Expression With OS

**Figure 3:**
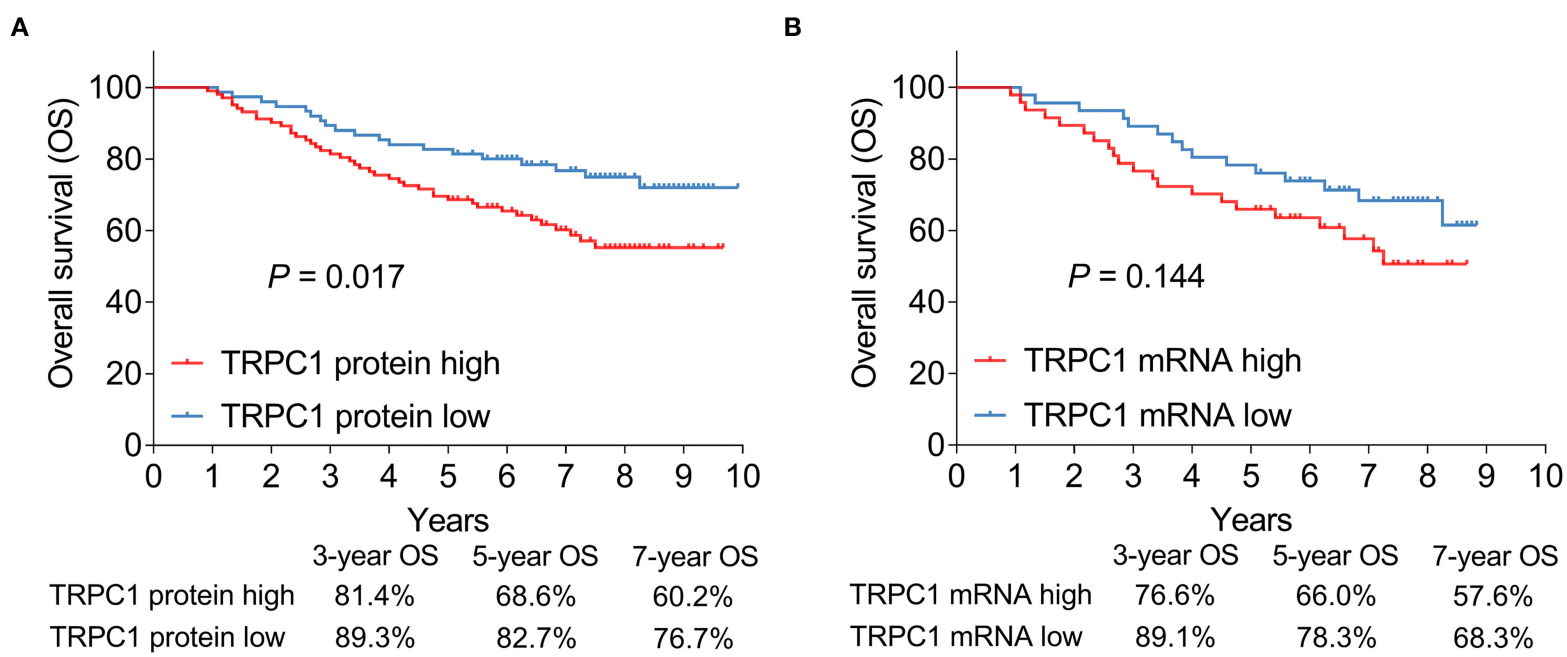
Association of tumor TRPC1 expression with prognosis in patients with RCC. Association of TRPC1 protein expression **(A)** and TRPC1 mRNA expression **(B)** with overall survival (OS). TRPC1, transient receptor potential channel 1; OS, overall survival; mRNA, messenger RNA; RCC, renal cell carcinoma.

The 3-, 5-, and 7-year OS were 81.4, 68.6, and 60.2%, respectively in patients with high TRPC1 protein,; as well as 89.3, 82.7, and 76.7%, respectively in patients with low TRPC1 protein. After statistical analysis, TRPC1 protein high (vs. low) was associated with poor OS (*P* = 0.017) (Figure 3A). Simultaneously, the 3-, 5-, and 7-year OS were 76.6, 66., and 57.6%, respectively in patients with high TRPC1 mRNA,; as well as 89.1, 78.3, and 68.3%, respectively in patients with low TRPC1 mRNA. However, there was no association in TRPC1 mRNA expression with OS (*P* = 0.144) (Figure 3B).

Univariate Cox's regression analysis revealed that TRPC1 protein expression (high vs. low) (*P* = 0.019, hazard ratio (HR) = 1.912), age (≥ 60 vs. < 60 years) (*P* = 0.019, HR = 1.86), ECOG PS score (1 vs. 0) (*P* = 0.017, HR = 1.98), higher pathological grade (*P* < 0.001, HR = 2.867), tumor size (> 7 vs. ≤ 7 cm) (*P* < 0.001, HR = 2.999), higher T stage (*P* < 0.001, HR = 2.828), higher N stage (*P* < 0.001, HR = 6.495), and higher TNM stage (*P* < 0.001, HR = 2.995) were correlated with worse OS. Next, the multivariate Cox regression analysis was conducted. By enter method, TRPC1 was not independently correlated with OS (*P* = 0.264, HR = 1.407), while ECOG PS score (1 vs. 0) (*P* = 0.015, HR = 2.098), higher pathological grade (*P* < 0.001, HR = 2.497), higher T stage (*P* = 0.012, HR = 2.431), and higher N stage (*P* < 0.001, HR = 6.129) were all independently associated with shorter OS. By forward stepwise method, TRPC1 protein expression (high vs. low) was independently correlated with poor OS (*P* = 0.01, HR = 2.052); other independent factors for the poor OS including ECOG PS score (1 vs. 0) (*P* = 0.007, HR = 2.196) and higher pathological grade (*P* < 0.001, HR = 3.066) (Table 2).

**Table 2:** Factors affecting overall survival (OS) by Cox's proportional hazards regression analysis.

**Items**	**Univariate Cox's regression analysis**		**Multivariable Cox's regression analysis (Enter)**		**Multivariable Cox's regression analysis (Forward stepwise)**	
	***P*-value**	**HR (95%CI)**	***P*-value**	**HR (95%CI)**	***P*-value**	**HR (95%CI)**
TRPC1 protein expression (high vs. low)	**0.019**	1.912 (1.110–3.293)	0.264	1.407 (0.772–2.564)	**0.010**	2.052 (1.189–3.541)
TRPC1 mRNA expression (high vs. low)	0.149	1.632 (0.840–3.173)	-	-	-	-
Age (≥ 60 years vs. < 60 years)	**0.019**	1.860 (1.108–3.125)	0.781	1.086 (0.606–1.949)	-	-
Gender (male vs. female)	0.997	0.999 (0.581–1.717)	0.803	1.077 (0.602–1.926)	-	-
ECOG PS score (1 vs. 0)	**0.017**	1.980 (1.131–3.468)	**0.015**	2.098 (1.158–3.802)	**0.007**	2.196 (1.244–3.880)
Tumor location (left vs. right)	0.144	1.463 (0.878–2.437)	0.463	1.233 (0.705–2.155)	-	-
Higher pathological grade	**<0.001**	2.867 (1.963–4.187)	**<0.001**	2.497 (1.594–3.912)	**<0.001**	3.066 (2.088–4.502)
Tumor size (> 7 cm vs. ≤ 7 cm)	**<0.001**	2.999 (1.810–4.967)	0.327	0.624 (0.244–1.601)	-	-
Higher T stage	**<0.001**	2.828 (1.970–4.060)	**0.012**	2.431 (1.213–4.874)	-	-
Higher N stage	**<0.001**	6.495 (3.521–11.980)	**<0.001**	6.129 (3.068–12.243)	-	-
Higher TNM stage	**<0.001**	2.995 (2.192–4.091)	-	-	-	-

*OS, overall survival; HR, hazard ratio; CI, confidence interval; TRPC1, transient receptor potential channel 1; ECOG PS, Eastern Cooperative Oncology Group Performance Status; TNM, tumor-node-metastasis*.

## Discussion

Several previous studies have reported that TRPC1 expression is increased in carcinoma tissue than in adjacent tissue of several cancers. For example, expression of TRPC1 in breast cancer tissue is higher than that in adjacent normal tissue (14, 19), in which TRPC1 is increased in colorectal cancer (CRC) tissues compared with adjacent normal tissues (20). In line with previous studies, our study found that TRPC1 expression was increased in the RCC tissue than in the adjacent tissue. A possible reason might be that TRPC1 represented a faster rate of cell proliferation, and the proliferation speed was higher in the RCC cells compared to the cells in adjacent tissues. Thus, TRPC1 expression was increased in the RCC tissue than in the adjacent tissue.

Regarding the correlation of TRPC1 expression with clinical features of patients with cancer, it has been elucidated that TRPC1 expression is positively associated with the TNM stage in breast cancer (14), whereas dysregulation of TRPC1 expression is associated with lymph node metastasis and differentiation in patients with ESCC (15). In our study, we observed that TRPC1 expression in the tumor was associated with higher T stage and TNM stage. The explanation for our findings was that TRPC1 might promote proliferation *via* Ca^2+^ entry and Ca^2+^- nuclear factor of activated T cells, cytoplasmic 3 (Ca^2+^-NFATc3) signaling pathways (12, 18), thus resulting in the RCC growth and subsequently affecting tumor size. Therefore, TRPC1 expression was correlated with elevated T stage and TNM stage. However, TRPC1 might have no capability of regulating invasion or stemness, thus no association was found in TRPC1 with N stage or differentiation.

In terms of the association of TRPC1 expression with prognosis in cancers, a preceding study shows that the dysregulation of TRPC1 is associated with poor prognosis in ESCC (15). Another study reveals that TRPC1 can serve as a prognostic biomarker in patients with breast cancer (13). The present study discovered that TRPC1 protein high was associated with an unsatisfactory survival in patients with RCC. Possible explanations could be the following: (1) TRPC1 expression was correlated with more advanced T stage and TNM stage, which might indirectly bring in shorter OS; (2) TRPC1 related to high EMT and invasive ability of RCC cells, which is associated with a high relapse risk, thus TRPC1 protein high was correlated with poor survival profile (12). Notably, TRPC1 was not an independent factor for OS by the enter method, which could be due to TRPC1 being correlated with T stage and TNM stage. Meanwhile, T stage and TNM stage were core factors influencing OS in our study. Thus, when TRPC1, T stage, and TNM stage were added in the multivariate Cox's regression analysis simultaneously, the prognostic value of TRPC1 was weakened. However, when the forward stepwise method was adopted, TRPC1 was an independent factor for worse OS. It should be noted that based on clinical experience, the most predominant factors affecting the prognosis of patients are disease stage and pathological differentiation. Biomarkers could be used to further help improve the prognostication of RCC. In the current study, no patients receive adjuvant therapy since according to the guideline from the Chinese Society of Clinical Oncology (CSCO), and since adjuvant therapy is not recommended for RCC patients because the benefit of adjuvant therapy is doubted in RCC.

It was worth mentioning that we have recorded the long-term follow-up data of the patients with RCC, which was an advantage for evaluating the prognostic value of TRPC1. However, the following limitations still existed. Firstly, to avoid possible interference, we only included clear cell subtype RCC, while the role of TRPC1 in patients with other RCC subtypes was not assessed. Secondly, this study might have relatively low statistical power due to the relatively small sample size. Thirdly, many patients in our study received surgery a long time (more than 5 years) ago. At that time, sample management in the hospital was not standard, and thus many liquid nitrogen-preserved fresh tissues were not available. Therefore, few samples could be used for RT-qPCR detection in our study. Fourthly, it was not easy to assess disease-free survival due to the excessively long follow-up duration of non-local patients. Fifthly, the proportion of patients with T1 stage was relatively high in the current study. Therefore, the findings of our study should be further verified in patients with T2 stage or above.

Conclusively, TRPC1 expression elevates in tumor tissues, correlates with more advanced T stage, TNM stage, and unsatisfactory long-term OS in patients with RCC. Meanwhile, TRPC1 could be served as a potential biomarker for RCC prognostication, which helps improve the management of patients with RCC.

## Data Availability Statement

The original contributions presented in the study are included in the article/Supplementary Material, further inquiries can be directed to the corresponding author.

## Ethics Statement

The studies involving human participants were reviewed and approved by Institutional Review Board of Renmin Hospital of Wuhan University. The patients/participants provided their written informed consent to participate in this study.

## Author Contributions

LC designed the study. LC, GS, MG, HQ, and YX acquired the data. LC and YX analyzed the data, edited the English language, and agreed to be accountable for all aspects of the work in ensuring that questions related to the accuracy or integrity of any part of the work are appropriately investigated and resolved. LC, GS, MG, and HQ drafted the manuscript. All authors read and approved the final manuscript.

## Conflict of Interest

The authors declare that the research was conducted in the absence of any commercial or financial relationships that could be construed as a potential conflict of interest.

## Publisher's Note

All claims expressed in this article are solely those of the authors and do not necessarily represent those of their affiliated organizations, or those of the publisher, the editors and the reviewers. Any product that may be evaluated in this article, or claim that may be made by its manufacturer, is not guaranteed or endorsed by the publisher.

## Supplementary Material

Supplementary Figure 1 Correlation between transient receptor potential channel (TRPC1) mRNA level and TRPC1 protein expression. Correlation between TRPC1 mRNA expression and TRPC1 immunohistochemistry (IHC) score in tumor tissues **(A)** and adjacent tissues **(B)**. TRPC1, transient receptor potential channel 1; IHC, immunohistochemistry; mRNA, messenger RNA.
