# Supplementary figures and images for: Transient Receptor Potential Channel 1 Potentially Serves as a Biomarker Indicating T/TNM Stages and Predicting Long-Term Prognosis in Patients With Renal Cell Carcinoma
Source: Front Surg. 2022 Apr 25;9:853310. doi: 10.3389/fsurg.2022.853310 (PMC9081676; doi:10.3389/fsurg.2022.853310)

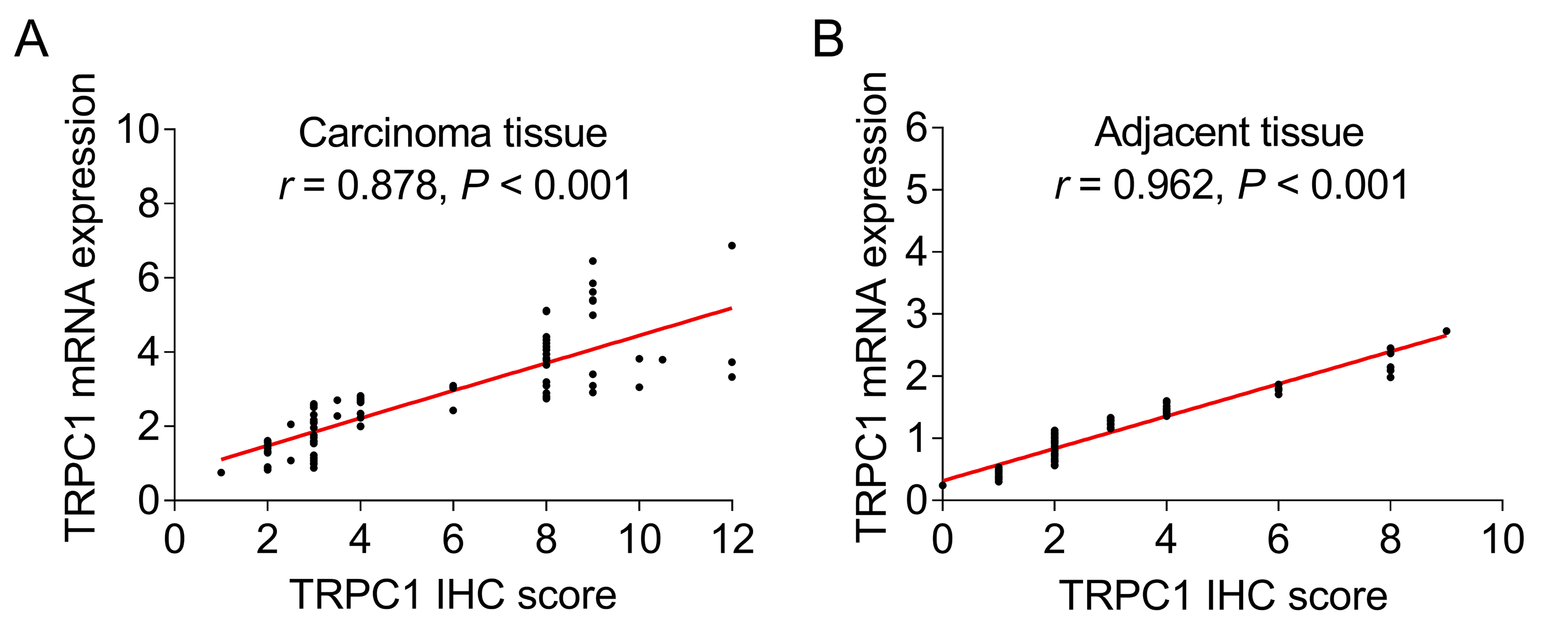

Supplement: Supplementary Figure 1 — Correlation between transient receptor potential channel (TRPC1) mRNA level and TRPC1 protein expression. Correlation between TRPC1 mRNA expression and TRPC1 immunohistochemistry (IHC) score in tumor tissues (A) and adjacent tissues (B). TRPC1, transient receptor potential channel 1; IHC, immunohistochemistry; mRNA, messenger RNA. [file Image_1.TIF]
